# Evaluation of pharyngeal airway space after orthodontic extraction treatment in class II malocclusion integrating with the subjective sleep quality assessment

**DOI:** 10.1038/s41598-023-36467-9

**Published:** 2023-06-06

**Authors:** Weerayuth Vejwarakul, Ellen Wen-Ching Ko, Cheng-Hui Lin

**Affiliations:** 1 Graduate Institute of Dental and Craniofacial Science, Chang Gung University, Taoyuan, Taiwan; 2 Department of Craniofacial Orthodontics, Chang Gung Memorial Hospital, 6F, 199, Tung Hwa North Road, Taipei, 105 Taiwan; 3 Craniofacial Research Center, Chang Gung Memorial Hospital, Linkou, Taoyuan, Taiwan; 4 Department of Plastic and Reconstructive Surgery, Chang Gung Memorial Hospital, Taoyuan, Taiwan

**Keywords:** Risk factors, Medical research, Outcomes research

## Abstract

Orthodontic treatment with premolar extractions is typically used to relieve dental crowding and retract anterior teeth for lip profile improvement. The aim of the study is to compare the changes in regional pharyngeal airway space (PAS) after orthodontic treatment with Class II malocclusion and to identify the correlations between questionnaire results and PAS dimensions after orthodontic treatment. In this retrospective cohort study, 79 consecutive patients were divided into normodivergent nonextraction, normodivergent extraction, and hyperdivergent extraction groups. Serial lateral cephalograms were used to evaluate the patients’ PASs and hyoid bone positions. The Pittsburgh Sleep Quality Index and STOP-Bang questionnaire were used for sleep quality evaluation and obstructive sleep apnea (OSA) risk assessment, respectively, after treatment. The greatest airway reduction was observed in hyperdivergent extraction group. However, the changes in PAS and hyoid positions did not differ significantly among three groups. According to questionnaire results, all three groups had high sleep quality and low risk of OSA, with no significant intergroup differences. Moreover, pretreatment-to-posttreatment changes in PAS were not correlated with sleep quality or risk of OSA. Orthodontic retraction with premolar extractions nither exhibit significant reduction in airway dimensions nor increase their risk of OSA.

## Introduction

In orthodontic camouflage treatment, premolar extractions are typically used to create space for the alignment of crowded teeth and correct the anteroposterior (AP) interarch discrepancies (including anterior teeth retraction) for achieving proper occlusion as well as improving the soft tissue and lip profile^1^. However, the health effects of obstructive sleep apnea (OSA) are a common concern among dental professionals, and some researchers have proposed that alteration of AP dimensions due to the extraction of premolars may increase a patient’s risk of OSA^2,3^.

The effects of orthodontic extraction on the pharyngeal airway space in adults was first investigated in 2010^2^. In recent years, several researches have been conducted on this topic, which have employed various imaging modalities and subject groups. However, the results of these studies are controversial, and the topic remains of considerable scholarly interest. Some studies have reported reductions in the airway space after orthodontic extraction^2–4^; others studies have reported no such changes^5–7^. Some researchers that have reported such reductions have attributed them to the retraction of incisors after premolar extraction, which decreases the arch length and volume of the oral cavity, leading to restriction of the tongue space and more posterior displacement of the tongue and soft palate^4^.

The evaluation of the pharyngeal airway was varied by the method of assessment^8^. In previous orthodontic studies, morphological changes in the airway have been assessed using two- or three-dimensional radiographic images. Lateral cephalograms, which are routine orthodontic radiographs, provide two-dimensional reconstructions of three-dimensional structures; therefore, the information that can be extracted from such images is limited^9^. However, lateral cephalograms remain widely used because of their widespread familiarity, simplicity, and low cost. Although cone-beam computed tomography (CBCT) is superior to lateral cephalograms for the assessment of the pharyngeal airway^9,10^, it has some limitations when used for this purpose, including the higher radiation dose, higher cost, and inherent nature as static imaging of a dynamic structure^11^.

Previous studies have mainly focused on morphological changes in the airway, and subjective sleep quality assessment has not been thoroughly investigated. Investigating the effects of airway dimensional changes on sleep quality and risk of OSA in healthy populations is crucial. Functional airway assessment through polysomnography (PSG) is a diagnostic gold standard, but PSG is not widely available^12^. Questionnaires can serve as effective, inexpensive, and rapid tools for the diagnosis of sleep disorders^13^. The STOP-Bang questionnaire (SBQ) assesses a set of four symptoms and four signs. A previous meta-analysis reported that the SBQ was superior to other tools for detecting mild, moderate, and severe OSA with high sensitivity^14^. The Pittsburgh Sleep Quality Index (PSQI) has been widely used in clinical practice as well as in studies as a tool for assessing the sleep quality of patients^15^. To the best of our knowledge, this is the first study to analyze and integrate data on morphological changes in the airway and the results of subjective sleep quality assessment conducted using two questionnaires.

Most previous studies have evaluated pharyngeal airway changes after orthodontic extraction treatment with maximum anchorage in patients with bimaxillary protrusion without skeletal discrepancy^2,3,5^. Few studies have focused on patients with Class II malocclusion^7,16^. The pharyngeal airway is affected by different craniofacial skeletal patterns, especially in patients with Class II and hyperdivergent skeletal patterns^17,18^. Moreover, a higher prevalence of mouth breathing and a higher risk of OSA have been reported in patients with Class II malocclusion^19,20^. To elucidate these findings, our study investigated the null hypothesis that the pharyngeal airway spaces of the nonextraction and extraction groups after orthodontic treatment would not differ.

The purposes of this study were as follows: (1) to compare the sagittal dimensional changes in regional pharyngeal airway spaces after orthodontic treatment among three groups of adult patients with Class II malocclusion (normodivergent nonextraction, normodivergent extraction, and hyperdivergent extraction groups) by using lateral cephalometric radiographs and (2) to identify correlations between the results of two questionnaires (the PSQI and SBQ) and data on airway space dimensions after orthodontic treatment.

## Results

### Differences in demographic characteristics

As listed in Table 1, the normodivergent nonextraction (control), normodivergent extraction, and hyperdivergent extraction groups consisted of 30 patients (7 men and 23 women; mean age, 24.52 ± 5.68 years; mean BMI, 20.57 ± 2.01 kg/m^2^), 23 patients (5 men and 18 women; mean age, 22.89 ± 5.54 years; mean BMI, 20.34 ± 2.34 kg/m^2^), and 26 patients (2 men and 24 women; mean age, 22.77 ± 4.98 years; mean BMI, 20.80 ± 2.57 kg/m^2^), respectively. The extraction groups contained higher proportions of patients Class II sagittal skeletal patterns than did the nonextraction group. No statistically significant differences were observed in sex distribution, mean age, or mean BMI among the three groups.

**Table 1:** Demographic characteristics of patients.

Variables	Normodivergent nonextraction (n = 30)	Normodivergent extraction (n = 23)	Hyperdivergent extraction (n = 26)	*p*-value
Gender, male:female	7:23	5:18	2:24	NS^ғ^
Age (years) (range of age)	24.52 ± 5.68 (18.35–35.71)	22.89 ± 5.54 (18.21–38.91)	22.77 ± 4.98 (18.03–36.64)	NS^†^
Body mass index (kg/m^2^)	20.57 ± 2.01	20.34 ± 2.34	20.80 ± 2.57	NS^†^
Treatment duration (years)	2.45 ± 0.87	2.95 ± 0.83	2.94 ± 0.84	0.049^†^*
Skeletal pattern				
Class I:Class II	15:15	9:14	8:18	NS^₭^
ANB (°)	5.77 ± 2.20	6.32 ± 2.57	6.83 ± 2.75	NS^†^
SN-MP (°)	32.60 ± 3.48	33.33 ± 3.36	45.28 ± 4.36	< 0.001^†^**
Extraction pattern, 2 or 4 premolars	0 : 30	3 : 20	1 : 25	-

*NS* not significant.

*p < 0.05, **p < 0.001.

*P* values calculated using ^ғ^Fisher exact test, ^†^one-way ANOVA, ^₭^Chi-square test.

The mean treatment durations of the normodivergent extraction and hyperdivergent extraction groups (2.95 ± 0.83 and 2.94 ± 0.84 years, respectively) were significantly longer than that of the nonextraction group (2.45 ± 0.87 years; *p* < 0.05).

### Intergroup differences in pretreatment variables

Cephalometric measurements at baseline (T0) among the groups were statistically analyzed (Tables 2 and 3). The mean values of 17 of the dentoskeletal parameters (except ANB, U1-SN, U6-X, and L6-X) differed significantly. Among the five soft tissue parameters, only mean Sn-Pog values differed significantly among the groups. No significant intergroup differences in pharyngeal airway dimensions or hyoid bone positions were identified.

**Table 2:** Pretreatment-to-posttreatment changes of skeletal and dental variables.

Variables	Normodivergent nonextraction							Normodivergent extraction							Hyperdivergent extraction							*p*-value^‡^		*p*-value^ғ^	
	T0		T1		∆T	SD	*p*-value^†^	T0		T1		∆T	SD	*p*-value^†^	T0		T1		∆T	SD	*p*-value^†^				
	Mean	SD	Mean	SD				Mean	SD	Mean	SD				Mean	SD	Mean	SD							
Skeletal variables																									
SNA	84.98	4.04	84.98	4.05	0.00	0.02	NS	84.64	3.42	84.61	3.41	-0.03	0.17	NS	81.45	3.32	81.43	3.31	-0.02	0.08	NS	0.001*		NS	
SNB	79.22	3.49	78.98	3.47	-0.24	0.79	NS	78.32	3.04	77.92	2.86	-0.40	0.80	0.025*	74.61	3.77	74.16	3.69	-0.45	0.60	0.001*	0.000**		NS	
ANB	5.77	2.20	6.01	2.37	0.24	0.80	NS	6.32	2.57	6.70	2.63	0.37	0.82	0.040*	6.83	2.75	7.26	2.75	0.43	0.60	0.001*	NS		NS	
SN-MP	32.60	3.48	32.71	3.69	0.10	1.37	NS	33.33	3.36	33.37	3.93	0.04	1.38	NS	45.28	4.36	45.18	4.43	-0.10	1.08	NS	0.000**		NS	
A-X	54.64	2.89	54.65	2.89	0.01	0.04	NS	53.04	2.66	53.06	2.62	0.02	0.10	NS	55.48	2.45	55.46	2.40	-0.02	0.16	NS	0.008*		NS	
B-X	96.84	4.42	97.38	4.55	0.55	1.63	NS	95.45	4.50	95.70	4.64	0.25	0.96	NS	100.02	4.61	99.74	4.34	-0.28	0.94	NS	0.002*		NS	
Pog-X	110.80	4.87	111.23	5.12	0.43	1.83	NS	108.83	5.71	109.71	6.20	0.88	1.37	0.006*	114.29	4.99	114.08	4.74	-0.21	1.16	NS	0.001*		0.043*	
A-Y	70.63	6.05	70.63	6.05	0.00	0.03	NS	69.32	3.50	69.32	3.51	0.00	0.07	NS	64.43	4.78	64.43	4.78	0.00	0.11	NS	0.000**		NS	
B-Y	60.84	7.82	60.35	7.89	-0.50	1.56	NS	58.45	4.17	57.74	4.34	-0.71	1.47	0.031*	49.55	7.81	48.74	7.63	-0.81	1.19	0.002*	0.000**		NS	
Pog-Y	60.82	9.24	60.12	9.11	-0.70	1.81	0.043*	57.19	4.84	56.50	5.02	-0.69	1.82	NS	46.32	8.89	45.77	8.81	-0.55	1.23	0.030*	0.000**		NS	
Head angulation	104.34	6.72	104.76	6.46	0.42	3.90	NS	105.33	6.16	104.91	3.88	-0.42	5.09	NS	110.03	6.50	111.82	7.82	1.78	4.28	0.044*	0.004*		NS	
Dental variables																									
U1-SN	106.71	10.26	105.59	5.88	-1.12	7.64	NS	110.85	7.62	98.89	4.96	-11.97	7.25	0.000**	106.42	8.54	95.62	5.72	-10.81	6.31	0.000**	NS		0.000**	
L1-MP	96.60	7.36	100.69	6.64	4.10	4.51	0.000**	101.33	6.42	94.70	8.00	-6.63	5.46	0.000**	92.98	6.65	86.91	5.82	-6.07	5.15	0.000**	0.000**		0.000**	
U1-X	80.83	3.68	80.39	3.83	-0.44	1.68	NS	79.46	4.25	78.58	3.43	-0.88	2.07	NS	83.15	3.60	82.02	2.87	-1.12	1.88	0.005*	0.004*		NS	
U1-Y	74.36	7.43	72.33	6.44	-2.03	2.73	0.000**	74.94	4.61	67.91	3.98	-7.03	3.10	0.000**	68.32	6.86	61.49	5.38	-6.83	2.71	0.000**	0.001*		0.000**	
L1-X	76.95	4.08	78.96	3.44	2.01	2.38	0.000**	75.91	3.81	77.17	3.50	1.26	1.67	0.002*	80.86	4.16	80.18	2.93	-0.68	2.45	NS	0.000**		0.000**	
L1-Y	69.55	6.60	69.64	6.20	0.08	1.71	NS	69.60	3.84	65.31	3.98	-4.29	2.78	0.000**	63.18	6.33	58.80	5.33	-4.39	2.41	0.000**	0.000**		0.000**	
U6-X	73.10	3.72	73.28	3.62	0.18	0.80	NS	71.23	3.04	71.80	3.21	0.57	0.63	0.000**	72.93	3.61	73.38	3.07	0.45	1.07	0.042*	NS		NS	
U6-Y	39.73	5.96	38.99	5.49	-0.74	1.72	0.025*	39.40	4.11	40.90	4.01	1.50	1.36	0.000**	34.85	5.50	36.37	5.01	1.52	1.99	0.001*	0.002*		0.000**	
L6-X	73.39	3.68	73.27	3.57	-0.12	0.95	NS	71.09	3.37	71.74	3.30	0.65	0.80	0.001*	72.75	3.74	73.18	3.21	0.43	1.04	0.043*	NS		0.011*	
L6-Y	38.87	6.09	38.79	5.60	-0.08	1.64	NS	37.12	4.74	39.57	4.23	2.46	2.36	0.000**	32.62	5.09	35.21	4.92	2.59	1.51	0.000**	0.000**		0.000**	

*NS* not significant.

*p < 0.05, **p < 0.001.

^†^Paired *t* test was performed for pretreatment-to-posttreatment changes (T0 to T1) within groups.

^‡^One-way ANOVA was performed for intergroup differences in pretreatment (T0) variables.

^ғ^One-way ANOVA was performed for intergroup differences in pretreatment-to-posttreatment changes (∆T).

**Table 3:** Pretreatment-to-posttreatment changes of soft tissue, airway and hyoid bone variables.

Variables	Normodivergent nonextraction							Normodivergent extraction							Hyperdivergent extraction							*p*-value^‡^	*p*-value^ғ^
	T0		T1		∆T	SD	*p*-value^†^	T0		T1		∆T	SD	*p*-value^†^	T0		T1		∆T	SD	*p*-value^†^		
	Mean	SD	Mean	SD				Mean	SD	Mean	SD				Mean	SD	Mean	SD					
Soft tissues																							
Uvula length	35.18	4.01	35.77	3.86	0.59	1.50	0.039*	35.74	4.20	36.31	3.59	0.57	1.32	NS	33.91	3.99	34.92	4.79	1.01	1.42	0.001*	NS	NS
Uvula angulation	126.16	6.04	127.18	5.79	1.02	2.95	NS	126.70	4.62	127.98	3.74	1.28	4.19	NS	125.57	5.55	127.07	5.41	1.51	2.97	0.016*	NS	NS
Sn-UL	4.55	2.14	3.17	2.33	-1.38	1.44	0.000**	5.37	2.01	1.85	2.11	-3.52	2.08	0.000**	4.20	1.96	0.90	2.40	-3.30	1.61	0.000**	NS	0.000**
Sn-LL	0.54	3.05	-0.10	3.14	-0.64	2.15	NS	2.18	2.82	-1.76	2.35	-3.93	2.60	0.000**	0.68	3.14	-3.47	2.80	-4.15	1.76	0.000**	NS	0.000**
Sn-Pog	-8.70	4.69	-8.73	5.19	-0.03	1.65	NS	-9.90	6.21	-10.61	4.63	-0.71	4.24	NS	-13.73	5.11	-14.20	4.89	-0.47	1.53	NS	0.002*	NS
Airway and hyoid bone variables																							
Airway1	24.19	3.17	24.02	2.72	-0.17	1.63	NS	24.07	2.87	24.40	2.62	0.32	1.05	NS	23.22	3.25	23.36	3.11	0.14	0.82	NS	NS	NS
Airway2	26.20	3.25	26.64	3.23	0.44	1.54	NS	26.62	2.90	27.14	3.00	0.53	1.46	NS	24.73	3.32	24.88	3.81	0.15	1.44	NS	NS	NS
Airway3	13.63	2.60	13.30	2.89	-0.34	1.58	NS	13.60	3.05	13.45	2.55	-0.16	1.38	NS	13.40	2.43	12.68	2.12	-0.72	1.91	NS	NS	NS
Airway4	11.63	2.45	11.52	2.41	-0.11	1.67	NS	11.67	2.82	11.24	2.65	-0.43	1.94	NS	11.13	3.03	10.53	2.87	-0.60	2.11	NS	NS	NS
Airway5	11.38	3.03	11.33	2.53	-0.06	2.01	NS	12.13	2.74	11.29	2.95	-0.84	2.40	NS	11.40	3.53	10.88	3.08	-0.52	2.27	NS	NS	NS
Airway6	16.89	2.93	17.39	2.68	0.50	1.46	NS	17.87	2.48	17.73	2.16	-0.14	2.03	NS	17.33	2.45	17.68	3.15	0.35	2.02	NS	NS	NS
H-RGN	36.16	5.47	36.17	5.95	0.01	3.64	NS	36.68	4.70	37.02	4.06	0.34	3.86	NS	34.02	4.11	34.32	5.97	0.29	3.93	NS	NS	NS
H-C3	35.86	3.54	36.63	3.68	0.77	1.70	0.020*	35.25	3.93	35.02	3.57	-0.23	1.88	NS	33.98	3.69	34.87	3.71	0.89	1.85	0.022*	NS	NS
H-MP	11.18	4.78	11.26	6.24	0.08	3.59	NS	11.26	6.17	11.61	6.06	0.35	3.66	NS	11.72	4.21	12.43	3.72	0.70	3.39	NS	NS	NS
H-S	110.23	9.42	110.94	10.06	0.71	3.50	NS	107.34	8.02	108.47	8.62	1.13	4.52	NS	105.06	7.03	106.09	5.93	1.03	3.93	NS	NS	NS

*NS* not significant.

*p < 0.05, **p < 0.001.

^†^Paired *t* test was performed for pretreatment-to-posttreatment changes (T0 to T1) within groups.

^‡^One-way ANOVA was performed for intergroup differences in pretreatment (T0) variables.

^ғ^One-way ANOVA was performed for intergroup differences in pretreatment-to-posttreatment changes (∆T).

### Pretreatment-to-posttreatment changes within groups

A comparison of pretreatment-to-posttreatment changes of each group is presented in Tables 2 and 3. No significant changes in pharyngeal airway dimensions or hyoid bone positions were observed, except for significant increases in HC3 in the normodivergent nonextraction and hyperdivergent extraction groups (*p* < 0.05). These increases indicated that after the treatment, the hyoid bone had moved anteriorly (although the movement was less than 1.00 mm).

### Intergroup differences in pretreatment-to-posttreatment changes (∆T)

No significant pretreatment-to-posttreatment changes in any of the skeletal variables were observed, except for a significant decrease in Pog-X in the hyperdivergent group (*p* < 0.05). Greater significant differences were observed in dental and soft tissue variables among the groups (*p* < 0.01). Upper and lower incisor retraction was more prominent in the extraction groups than in the nonextraction group (Table 2). In the extraction groups, the upper first molars had moved forward by 1.50 and 1.52 mm, and the lower first molar had moved forward 2.46 and 2.59 mm in normodivergent and hyperdivergent groups, respectively. Notably, however, no significant differences were identified in the changes in pharyngeal airway dimensions or hyoid bone positions among the groups.

For qualitative analysis, reductions in patients’ airway dimensions were measured individually (Table 4). The chi-square test revealed no significant differences in airway dimension reduction among the groups. Regarding the changes in airway dimensions by section, the sections most susceptible to dimensional reduction were those in the area behind the soft palate and tongue (sections 3, 4 and 5).

**Table 4:** Intergroup differences in pharyngeal airway changes (∆T) by airway section (sections 1–6).

Variables	Normodivergent nonextraction (n = 30)			Normodivergent extraction (n = 23)			Hyperdivergent extraction (n = 26)			*p-*value	Chi-square	Total (n = 79)	Cases with airway reduction (%)
	T1–T0		Count of airway reduction	T1–T0		Count of airway reduction	T1–T0		Count of airway reduction				
	Mean	SD		Mean	SD		Mean	SD					
Airway1	−0.17	1.63	15	0.32	1.05	10	0.14	0.82	10	NS	NS	0.07	35 (44.30%)
Airway2	0.44	1.54	13	0.53	1.46	8	0.15	1.44	12	NS	NS	0.37	33 (41.77%)
Airway3	−0.34	1.58	15	−0.16	1.38	12	−0.72	1.91	15	NS	NS	−0.41	42 (53.16%)
Airway4	−0.11	1.67	15	−0.43	1.94	14	−0.60	2.11	16	NS	NS	−0.36	45 (56.96%)
Airway5	−0.06	2.01	15	−0.84	2.40	14	−0.52	2.27	17	NS	NS	−0.44	46 (58.23%)
Airway6	0.50	1.46	13	−0.14	2.03	12	0.35	2.02	12	NS	NS	0.27	37 (46.83%)

One-way ANOVA was performed for intergroup differences in airway changes (∆T).

Chi-square test were performed for intergroup differences in count of airway reduction.

*NS* not significant.

According to a descriptive analysis (SI Table 1), the hyperdivergent extraction group exhibited the greatest total airway reduction (sum of changes in sections 1–6), with a mean reduction of − 0.20 ± 1.85 (corresponding to approximately 52.56% of the total airway).

### Relationships between changes in dentoskeletal, soft tissue, and hyoid bone variables and pharyngeal airway dimensional changes

In the multiple linear regression analysis (Table 5), pretreatment-to-posttreatment changes in pharyngeal airway dimensions were not significantly correlated with changes in skeletal variables or upper and lower incisor retraction. Interestingly, increases in airway section 5 (in the portion of the airway behind the tongue) were strongly associated with head angulation. A strong negative correlation was identified between uvula angulation and reduction in the portion of the airway at the tip of the soft palate. Moreover, increases in the movement of hyoid bone, in both horizontally (ΔH-RGN) and vertically (ΔH-MP) was positively correlated with the head angulation (SI Table 2).

**Table 5:** Multiple linear regression of relationships between pretreatment-to-posttreatment changes in dentoskeletal, soft tissue, and hyoid bone variables and pharyngeal airway dimensions.

Variables		ΔAirway1	ΔAirway2	ΔAirway3	ΔAirway4	ΔAirway5	ΔAirway6
ΔSNA	β	4.57	6.68	3.70	0.43	−3.61	−4.88
	*p*	0.349	0.162	0.487	0.927	0.537	0.341
ΔSNB	β	−5.53	−3.52	−2.51	−3.85	0.64	2.49
	*p*	0.257	0.457	0.636	0.417	0.912	0.625
ΔANB	β	−2.29	−4.29	0.37	-0.39	1.70	0.37
	*p*	0.597	0.313	0.938	0.927	0.744	0.935
ΔSN-MP	β	0.18	0.13	0.09	0.00	0.25	−0.23
	*p*	0.404	0.537	0.709	0.989	0.326	0.303
ΔHead angulation	β	0.02	0.09	0.08	0.22	0.41	0.27
	*p*	0.853	0.322	0.439	0.020*	0.000**	0.007*
ΔU1-Y	β	−0.29	−0.24	−0.27	−0.36	−0.31	−0.09
	*p*	0.123	0.185	0.187	0.054	0.177	0.647
ΔL1-Y	β	0.02	0.01	0.00	−0.10	0.14	−0.10
	*p*	0.924	0.97	0.998	0.628	0.575	0.632
ΔUvula length	β	0.09	0.13	−0.45	−0.52	−0.47	−0.15
	*p*	0.58	0.411	0.011*	0.001*	0.015*	0.374
ΔUvula angulation	β	0.02	0.08	−0.04	−0.29	−0.15	−0.10
	*p*	0.786	0.315	0.659	0.000**	0.103	0.218
ΔH-RGN	β	0.10	0.11	0.22	0.25	−0.06	−0.02
	*p*	0.416	0.369	0.117	0.044*	0.669	0.909
ΔH-C3	β	0.08	−0.09	−0.06	0.08	0.10	0.31
	*p*	0.539	0.472	0.691	0.517	0.528	0.027*
ΔH-MP	β	−0.29	−0.18	−0.25	-0.33	−0.03	−0.02
	*p*	0.145	0.344	0.253	0.086	0.897	0.919
ΔH-S	β	0.20	0.17	0.22	0.30	0.13	−0.02
	*p*	0.202	0.256	0.195	0.053	0.496	0.890

β, beta coefficient, indicates positive or negative correlations between variables of interest.

*p < 0.05, **p < 0.001.

∆ indicates pretreatment-to-posttreatment changes in variables of interest.

### Posttreatment sleep quality and OSA risk assessment

At posttreatment, questionnaires were administered to 65 of the patients (Table 6). The mean PSQI scores in the normodivergent nonextraction, normodivergent extraction, and hyperdivergent extraction groups were 4.00 ± 1.65, 3.90 ± 2.00, and 4.09 ± 2.18, respectively, indicating that all the groups had high sleep quality after orthodontic treatment. No significant intergroup differences were identified in the mean PSQI scores or in the proportion of patients with low sleep quality. The mean SBQ scores of the normodivergent nonextraction, normodivergent extraction, and hyperdivergent extraction groups were 0.43 ± 0.73, 0.30 ± 0.57, and 0.27 ± 0.55 respectively, indicating that all the groups were at low risk of OSA, with no significant intergroup differences.

**Table 6:** Sleep questionnaire results.

	Normodivergent nonextraction (n = 23)	Normodivergent extraction (n = 20)	Hyperdivergent extraction (n = 22)	Total (n = 65)	*p*-value
PSQI scores	4.00 ± 1.65	3.90 ± 2.00	4.09 ± 2.18	4.00 ± 1.92	NS^†^
Poor sleeper (%)	4	4	5	13 (20.00%)	NS^ғ^
SBQ scores	0.43 ± 0.73	0.30 ± 0.57	0.27 ± 0.55	0.34 ± 0.62	NS^†^
Intermediate risk (%)	1	0	0	1 (1.54%)	NS^ғ^
Snoring	1	1	1	3 (4.62%)	NS^ғ^
Tired	2	1	2	5 (7.69%)	NS^ғ^
Observed	0	0	0	0 (0.00%)	NS^ғ^
Pressure	0	0	0	0 (0.00%)	NS^ғ^

The *p* value calculated using ^†^one-way ANOVA, ^ғ^Fisher exact test.

*NS* not significant.

The descriptive statistics for the PSQI and SBQ are presented in Table 6. Among the 65 patients, three and five patients reported snoring and tiredness, respectively. None of the patients reported apnea (cessation of breathing) or high blood pressure after orthodontic treatment, and no significant intergroup differences were observed.

In the multiple logistic regression analysis (SI Table 3), pretreatment-to-posttreatment changes in pharyngeal airway dimensions were not significantly correlated with sleep quality or OSA risk.

## Discussion

The present study investigated the potential effects of premolar extraction on the pharyngeal airway. This is the first study to use the PSQI and SBQ as tools for subjective sleep quality assessment.

Regarding our methodology, all three groups revealed no differences in baseline demographic characteristics, which minimized the effect of confounding factors on the results. The normodivergent nonextraction group served as the control group for comparison with the experimental groups. Most previous studies have only analyzed pretreatment-to-posttreatment changes without including control groups, with a potential risk of bias for their conclusions^2–5^.

Although the patients’ pharyngeal airways were assessed using two-dimensional radiographs, which cannot capture 3-dimensional configurations, a recent study reported a strong correlation between pharyngeal airway area measurements on lateral cephalograms and the true volumetric data of the airways according to CBCT images^10,21^. One limitation of our study is that the pharyngeal airways were assessed only in the AP dimension; we did not account for cross-sectional dimensions or minimal cross-sectional area. By contrast, one strength of our study is that although previous studies have used two-dimensional radiographs for orthodontic evaluations^2,3,5^, only the present study conducted the calibration of 2D images by superimposing patients’ T0 and T1 cephalograms by using the anterior cranial base and frontonasal suture as reference structures before the evaluation of all variables of interest. This ensured accurate size calibration when using Dolphin Imaging software for landmark identification and measurements. Therefore, the errors from manual point selection and reconstruction were minimized.

In our study, no statistically significant difference was observed in the pharyngeal airway dimensional changes or hyoid bone positions between the extraction and nonextraction groups. This finding is in agreement with those of previous studies^5–7^; Paliska et al. and Joy et al. have also reported that the changes in pharyngeal airway volume and minimal cross-sectional area did not differ significantly between the nonextraction and extraction groups^6,7^.

In contrast to the findings of our study and the aforementioned studies, other studies have reported reductions in the pharyngeal airway space, especially in the glossopharyngeal area^3,4^. Zhang et al. had proposed that airways tend to self-regulate; that is, when an airway is narrow in the AP dimension, it expands in the lateral dimension to ensure sufficient space for air passage^16^. The pharyngeal airway seems to undergo adaptive morphological changes rather than decreasing in size; consequently, the airway volume, height, and cross-sectional area do not change significantly. Through computational fluid dynamics simulation, Zheng et al. also reported that the pressure drops in the oropharynx increased after premolar extraction^22^, indicating the collapse of the pharyngeal airway. However, all these studies accounted only for pretreatment-to-posttreatment changes without the inclusion of control groups, and the findings are therefore at risk of bias as well as errors associated with study design^3,4,22^.

The present study used the multiple linear regression to identify factors that may be correlated with airway dimensional changes, including dental features, skeletal changes, soft tissue parameters, and hyoid bone position-related variables.

Incisor retraction has been proposed to be related to airway reduction. Wang et al. illustrated that lower incisor retraction of 4.95 mm was negatively associated with the pharyngeal airway space^3^. Chen et al. revealed that upper incisor retraction of 7.64 mm negatively affected airway dimensions^4^. The authors attributed this finding to the reduction of pharyngeal airway dimensions resulting from the amount of incisor retraction. In our study, the upper and lower incisors retracted by 7.03 and 4.29 mm, respectively, which are close to the aforementioned values in the previous studies^3,4^. However, our linear regression analysis revealed that neither changes in dentoskeletal parameters nor incisor retraction were correlated with airway dimensional changes, which is agreement with the results of 2 previous studies^16,23^.

Difference in vertical skeletal patterns have been reported to be related to differences in pharyngeal airway dimensions^17,18^. Patients with hyperdivergent facial patterns tend to have smaller airways^18^. In the present study, the dimensions of each section of the airway at baseline (T0), the differences were not statistically significant. The effect of premolar extraction on airway dimensions may be stronger among patients with hyperdivergent facial types, as reported in a previous study^23^. Furthermore, the hyperdivergent extraction group exhibited the greatest reduction in airway dimensions, although the intergroup difference was statistically nonsignificant. Hyperdivergence may have an impact on the airway given the down and backward position of the mandible and other hard tissue attachments to the airway-associated structures. This may indicate that extraction exerts a greater negative effect on the airway in patients with hyperdivergent facial patterns.

More importantly, our findings demonstrate the importance of controlling the facial vertical dimensions after orthodontic treatment. The pretreatment-to-posttreatment increase in vertical dimensions (FH-MP increased by 0.43°) exerted a negative effect (*p* < 0.01) on the dimensions of the portion of the airway behind the soft palate and tongue^23^, which is in conflict with the findings of our study and two previous studies,^6,16^ the patients’ facial vertical dimensions were maintained after treatment; consequently, no drastic changes in their pharyngeal airway dimensions were noted.

The hyoid bone position is usually used to determine the position of the tongue^24,25^. In the present study, the horizontal and vertical movements of tongue were measured according to the most superior and anterior points of the hyoid bone, and its projections toward those skeletal landmarks. The different results of hyoid bone movement following an orthodontic treatment have been reported by previous studies^4,16^, possibly because of the various attachment of certain muscles insert to its free bone body. In our study, on average, patients’ hyoid bones moved anteriorly and inferiorly by clinically nonsignificant amounts (0.89 mm and 0.70 mm, respectively).

According to our linear regression analysis, the hyoid bone position was affected only by changes in head angulation. This result indicates that it is more strongly influenced by skeletal positional changes (such as changes in head posture or mandibular movement caused by orthognathic surgery^24,26^) than by dental positional changes and related factors, such as BMI and increases in neck circumference^27^. These findings suggest that incisor retraction and other dental positional changes do not exert any effect on changes in the tongue position and, in turn, may not be responsible for pharyngeal airway reduction.

Changes in head angulation have been identified to be related to changes in orthodontic treatment, including airway dimensions and hyoid bone positions^28,29^. In one study, increasing the craniocervical inclination in the second vertebrae by 10° increased the pharyngeal airway space at the glossopharynx by approximately 4 mm^29^. This is important because when airway dimensions are measured using two-dimensional radiographs, the effect of the head posture must be considered to determine the effects that can be accurately attributed to orthodontic treatment. In our study, the pretreatment-to-posttreatment changes in head angulation in all three groups were minimal and therefore may not have affected the patients’ airway dimensions.

To the best of our knowledge, this is the first study to conduct subjective airway assessment by using two questionnaires (the PSQI and SBQ). The PSQI reflects subjective sleep quality, habitual sleep efficiency, and sleep disturbances^15^. A previous meta-analysis showed that the SBQ is superior to other questionnaires for detecting OSA, with the highest sensitivity (87.0%) and moderate specificity (76.0%)^30,31^. In the present study, no statistically significant differences in questionnaire results were identified between the extraction and nonextraction groups; furthermore, 80% of the patients had high sleep quality, and 98.46% were at low risk of OSA.

By integrating cephalometric data, we determined that morphological changes in the airway were not correlated with sleep quality or risk of OSA after orthodontic treatment. This finding agrees with that of a previous study, in which electronic medical and dental health records were integrated with PSG data. The authors identified no relationship between OSA and premolar extraction^32^. In addition, a recent systematic review and meta-analysis reported no correlation between premolar extraction and changes in pharyngeal airway volume or minimum cross-sectional area^33^.

Despite our integration of cephalometric data and subjective airway assessment and standardization of 2D image acquisition, the present study still has some limitations. First, this was a retrospective study. Our findings should be further evaluated through randomized control trials, which are considered the gold standard in research design. Second, the transverse dimensions of patients’ airways could not be assessed. CT imaging should be used to overcome this limitation in future studies. Third, this study did not account for long-term changes after orthodontic treatment. Finally, although questionnaires are convenient tools for subjective airway assessment, PSG should be adopted in future investigations because it produces higher-quality results.

In conclusion, Orthodontic extraction treatment and incisor retraction do not affect pharyngeal airway dimensions. Vertical control of Class II skeletal malocclusion, especially in cases involving retrusive chins, can be applied to prevent worsening of the facial profile and to mitigate a tendency of reduction of airway dimensions. Orthodontic extraction treatments did not diminish the patients’ sleep quality or enhance their risk of OSA.

## Methods

### Participants

This retrospective cohort study included 79 consecutive Taiwanese patients who underwent orthodontic treatment from September 2010 to March 2021 at the Chang Gung Craniofacial Center and who met the following inclusion criteria: (1) age of 18 to 40 years; (2) dental Class II malocclusion or subdivision; and (3) no missing teeth (excluding the third molars). The exclusion criteria were as follows: (1) body mass index (BMI) ≥ 24 kg/m^2^; (2) previous orthodontic treatment; (3) previous treatment involving specific approaches (rapid maxillary expansion, functional appliances); (4) cleft lip or palate; (5) history of facial trauma or temporomandibular disorders; (6) history of orthognathic surgery; (7) hyperplasia of the tonsils or adenoids.

The sample size was calculated using G*Power software (version 3.1.9.4; Heinrich-Heine-Universität Düsseldorf, Düsseldorf, Germany). Based on the difference in sagittal airway dimensions at glossopharynx (TB-TPPW: TB, anterior pharyngeal wall at the point of tongue base; TPPW, point of intersection line from TB perpendicular to posterior pharyngeal wall) in a previous study^3^, the minimum sample size required to achieve 80% power and 95% confidence for the independent samples *t* test was 21 per group.

In this study, 79 patients were divided into 3 groups according to whether they received nonextraction or extraction treatment and their initial mandibular plane angle (SN-MP), with SN-MPs from 27° to 39° and more than 39° being categorized as normodivergent and hyperdivergent, respectively. Ultimately, three groups were established: (1) the normodivergent nonextraction (control) group, (2) the normodivergent extraction group, and (3) the hyperdivergent extraction group.

### Treatment protocols

All patients received orthodontic camouflage treatment provided by an orthodontist (E.W.C.K.) using 0.022 × 0.028-in. preadjusted edgewise brackets with sliding mechanics. The indications for premolars extraction were considered including space requirements for the alignment of crowded teeth, the correction of anteroposterior interarch discrepancy, dental arch width, patient’s soft tissues profile, and the patient’s age. In the extraction groups, the treatment mechanics were associated with maximum anchorage in the upper arch, and minimal-to-moderate anchorage in the lower arch. A treatment with the vertical controlling was considered, particularly in cases with skeletal Class II relationships, retrusive chin and a hyperdivergent facial pattern. To prevent the posttreatment increasing of vertical dimension, causing backward and downward movement of the mandible, careful use of Class II elastic was concerned. Moreover, the use of vertical controlling devices, such as transpalatal arch, temporary anchorage devices or other treatment approaches, were considered to use individually owing to the conditions of each patient.

This study was conducted in accordance with the medical protocols and ethical guidelines outlined in the Declaration of Helsinki, and the study methodology was approved by the Institutional Review Board of Chang Gung Memorial Hospital.

### Data collection

Serial lateral cephalograms were acquired in the natural head position without swallowing, and all the patients’ teeth were in centric occlusion. The images were obtained using a Gendex X-ray system (GXDP-700, Kavo Dental, Biberach, Germany) at pretreatment (T0) and posttreatment (T1). All the cephalograms were imported into Dolphin Imaging software (version 11.5; Major Partner SAS, Villanova d'Asti, Italy). Consequently, the actual size of each image in millimeters was calibrated according to a 10-mm ruler embedded on the cephalograms. This calibration method was used to standardize all the image size. The radiographs were evaluated by one investigator (V.W.).

### Cephalometric landmarks and measurements

**Figure 1:**
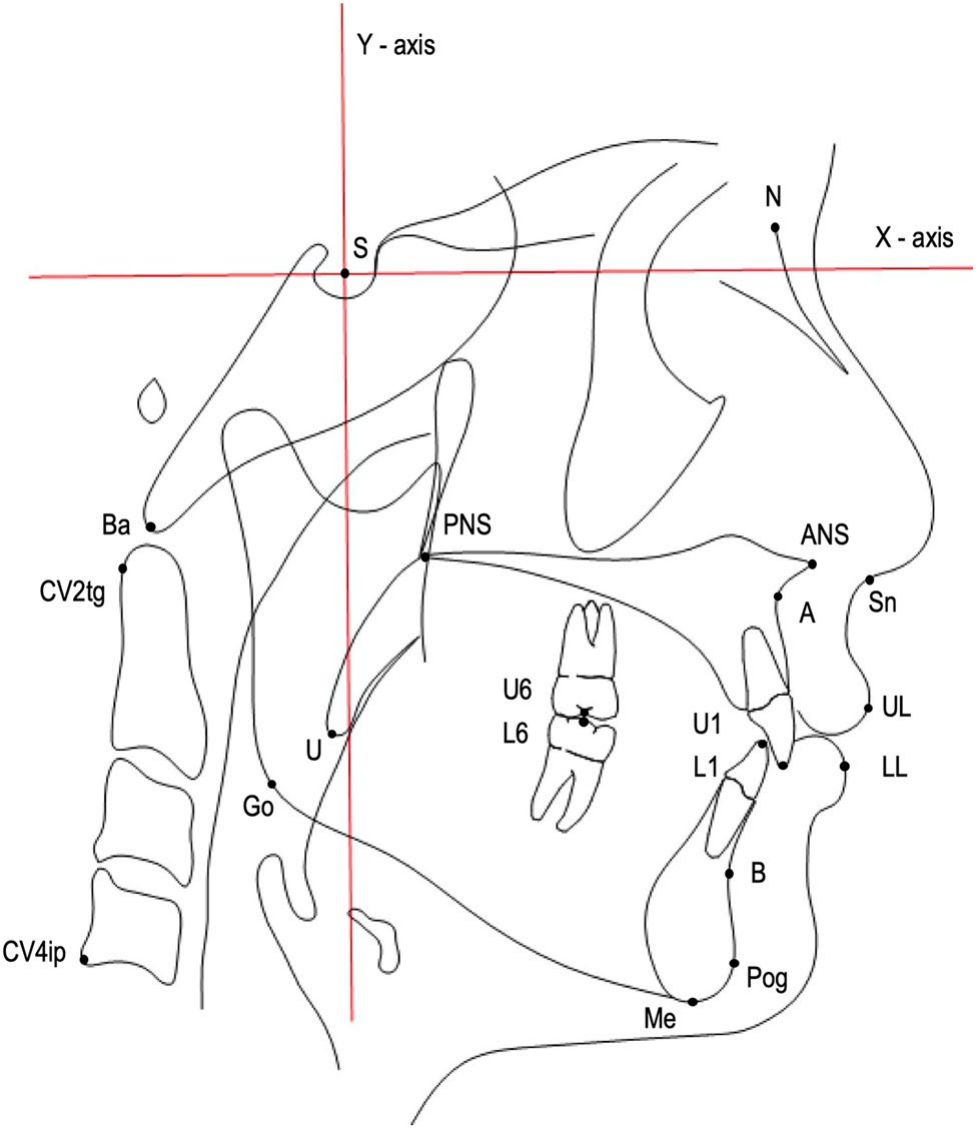
Cephalometric landmarks and measurements of dentoskeletal and soft tissue parameters. *S* sella, *N* nasion, *Ba* basion, *ANS* anterior nasal spine, *PNS* posterior nasal spine, *A* point A, *B* point B, *CV2tg* tangent point of the superior posterior extremity of the odontoid process of the second cervical vertebra, *CV4ip* the most inferior posterior point on the body of the fourth cervical vertebra, *U* uvula tip, *Go* gonion, *Me* menton, *Pog* pogonion, *Sn* subnasalae, *UL* upper lip, *LL* lower lip, *U1* tip of upper incisor, *L1* tip of lower incisor, *U6* midocclusal point of upper first molar, *L6* midocclusal point of lower first molar, *X-axis* 6° below SN line, *Y-axis* line perpendicular to X-axis through sella.

**Figure 2:**
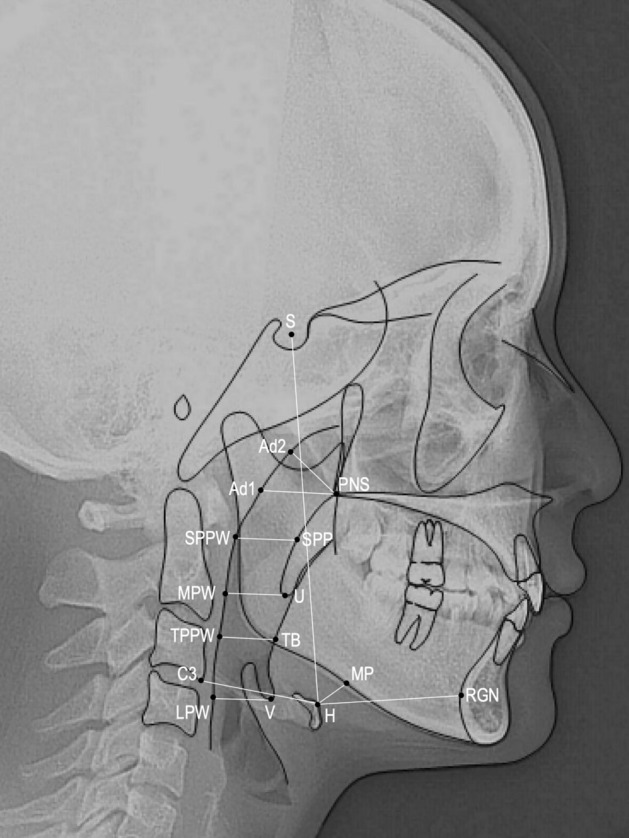
Cephalometric landmarks and measurements of pharyngeal airway dimensions and hyoid bone positions. *Ad1* point of posterior pharyngeal wall with intersection line from PNS to Ba, *Ad2* point of posterior pharyngeal wall with intersection line from PNS to midpoint of SBa line, *SPP* midposterior margin of soft palate, *SPPW* point of intersection line from SPP perpendicular to posterior pharyngeal wall, *MPW* point of intersection line from U perpendicular to posterior pharyngeal wall, *TB* point of tongue base from B-Go intersection line, *TPPW* point of intersection line from TB perpendicular to posterior pharyngeal wall, *V* most posteroinferior point on the base of the tongue, *LPW* point of intersection line from V perpendicular to posterior pharyngeal wall, *H* hyoid bone, *RGN* retrognathion, *MP* point of intersection line from H perpendicular to mandibular plane, *C3* the most inferior/anterior point on the body of the third cervical vertebra.

All available landmarks, lines, and measurements were constructed using Dolphin Imaging software for the custom analysis used in this study. The X-axis (6° below the SN line) and Y-axis (the line perpendicular to the X-axis through the sella) were set as the horizontal and vertical reference planes, respectively. The cephalometric measurements collected accounted for 36 variables, comprising 21 dentoskeletal parameters, 5 soft tissue variables, and 10 pharyngeal airway dimensions and hyoid bone positions. Measurements of the sagittal dimensions of the pharyngeal airway as well as the hyoid bone position were conducted according to the modification of a previously described method^3,23^. The sagittal dimensions of the pharyngeal airway were measured at six sections: two sections of the nasopharynx (PNS-Ad2 and PNS-Ad1), three sections of the oropharynx (SPP-SPPW and U-MPW in the velopharynx behind the soft palate and TB-TPPW in the glossopharynx behind the tongue base), and one section of the hypopharynx (V-LPW). In addition, the horizontal and vertical dimensions of the hyoid bone position were described in terms of H-RGN, H-C3, H-MP, and H-S. All landmarks, lines, and measurements used are presented in Figs. 1 and 2.

**Figure. 3:**
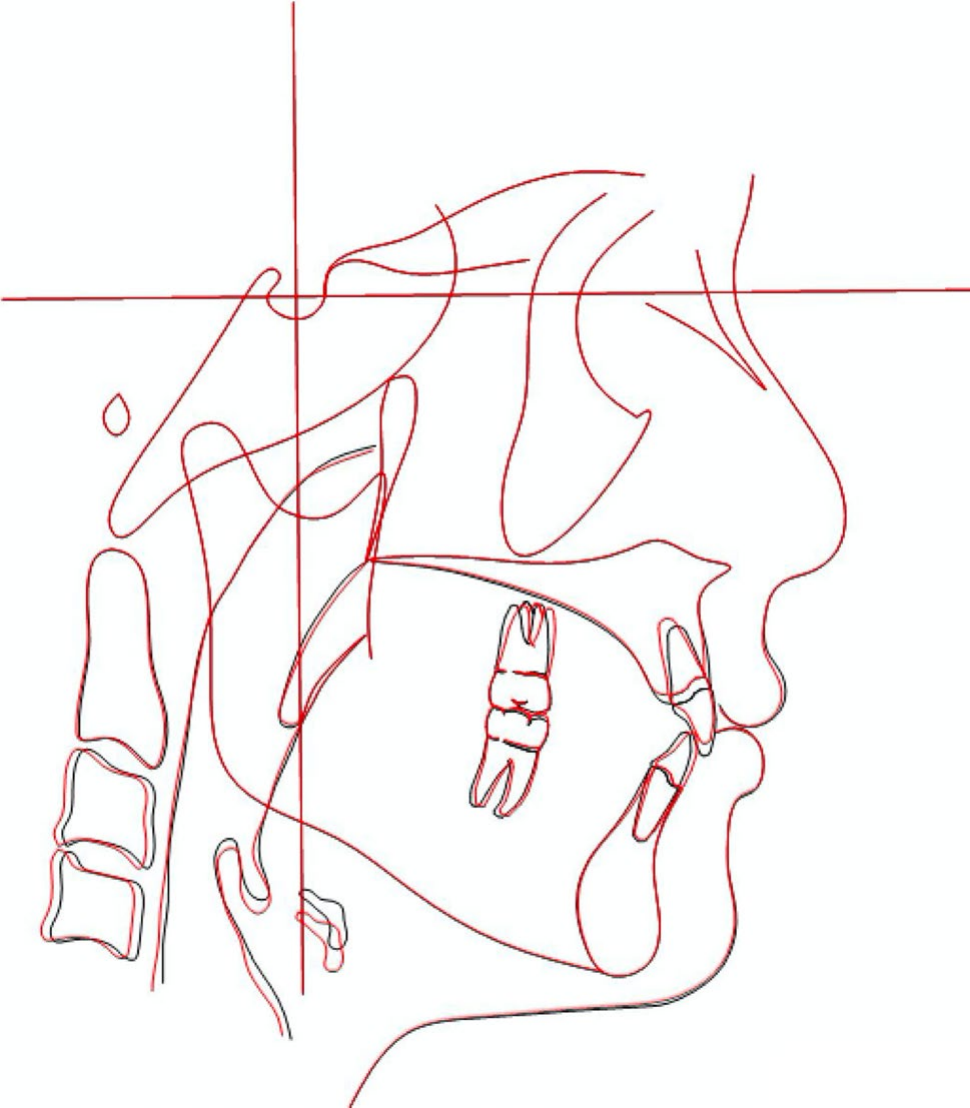
The standardization of 2D images acquisition by the superimposition of T0 (black line) and T1 (red line) cephalograms at anterior cranial base, frontonasal suture and cervical 2 (C2) vertebra as reference structures for registration.

Subsequently, for each patient, cephalograms obtained at T0 and T1 were superimposed using the anterior cranial base, frontonasal suture and cervical 2 (C2) vertebra as reference structures for registration before the evaluation of all the variables of interest (Fig. 3).

### Sleep quality and OSA risk assessment

Two questionnaires were administered in this study: the PSQI and the SBQ. Posttreatment data collection was conducted by one interviewer (Y.TL.). The total scores of the PSQI, which are used to assess the sleep quality of patients, range from 0 to 21. A total PSQI score of ≤ 5 indicates high sleep quality; a total PSQI score of > 5 indicates low sleep quality.

The SBQ was used to evaluate patients’ risk of OSA after orthodontic treatment. Total SBQ scores of 0 to 2, 3 to 4, and ≥ 5 indicate low, intermediate, and high risks of OSA, respectively. Patients with intermediate SBQ scores and at least one of the following contributing factors were considered to be at high risk: male sex, BMI > 35 kg/m^2^, and neck circumference > 40 cm.

### Statistical analysis

Statistical analyses were conducted using SPSS software (version 22.0, IBM, Chicago, IL, USA). The baseline (T0) demographic characteristics of the three groups were compared. Gender and the sagittal skeletal pattern (Class I or II) were analyzed using the Fisher exact test and the Chi-square test, respectively; one-way analysis of variance (ANOVA) was used for the assessment of age, BMI, and ANB and SN-MP angles.

A Shapiro–Wilk test and Levene test confirmed the normal distribution of the data and the homogeneity of the variances, respectively. Pretreatment-to-posttreatment changes (∆) were analyzed using a paired *t* test, and intergroup comparisons were conducted through one-way ANOVA. Multiple linear regression was used to identify any correlations between changes in dentoskeletal variables and changes in the pharyngeal airway. All data are expressed as means and standard deviations (SDs); a *p* value < 0.05 was considered statistically significant.

The questionnaire results are presented as descriptive statistics. Thereafter, the intergroup comparison was conducted through one-way ANOVA. Multiple logistic regression analysis was conducted to identify correlations between changes in the pharyngeal airway and posttreatment sleep quality and OSA risk.

To assess intra-examiner reliability, 15 randomly selected radiographs were retraced at 3-week intervals by the same investigator. All the cephalometric landmarks and measurements were re-evaluated. Intraclass correlation coefficients (ICCs) were used to assess both the consistency and agreement of the measurements between the two time points. The Dahlberg formula was used to evaluate linear and angular measurement errors. The errors of the linear and angular measurements ranged from 0.21 to 0.43 mm and 0.24° to 0.53°, respectively. Moreover, the ICC was 0.996 with a 95% confidence interval (0.991 to 0.999) indicating excellent reliability.

### Ethics approval

The research protocol was approved by the Institutional Review Board and Medical Ethics Committee of Chang Gung Memorial Hospital (No. 202101880B0).

### Informed consent

A written informed consent was obtained from all study participants.

## Supplementary Information

Supplementary Tables.

## Data Availability

Please contact corresponding author if someone wants to request the data for this study.
