# Supplementary material for: Evaluation of pharyngeal airway space after orthodontic extraction treatment in class II malocclusion integrating with the subjective sleep quality assessment
Source: Sci Rep. 2023 Jun 6;13:9210. doi: 10.1038/s41598-023-36467-9 (PMC10244355; doi:10.1038/s41598-023-36467-9)
Supplement: Supplementary file 1 — Supplementary Tables. [file 41598_2023_36467_MOESM1_ESM.docx]

**Supplementary Tables**

**Sl Table 1.** Descriptive statistics for total airway changes following orthodontic treatment.

| Variables | Total airway change | | 95% Confidence interval | | Cases with airway reduction  (%) |
| --- | --- | --- | --- | --- | --- |
|  | Mean | SD | Lower | Upper |  |
| **Normodivergent nonextraction** | 0.05 | 1.67 | -0.20 | 0.30 | 47.78% |
| **Normodivergent extraction** | -0.12 | 1.80 | -0.41 | 0.17 | 50.72% |
| **Hyperdivergent extraction** | -0.20 | 1.85 | -0.49 | 0.08 | 52.56% |

Total airway change was calculated by summing the changes in airway dimensions (∆T) for all 6 airway sections.

**Sl Table 2.** Multiple linear regression of relationships between pretreatment-to-posttreatment changes in dentoskeletal, soft tissue variables and hyoid bone positions.

| Variables |  | ΔH-RGN | ΔH-C3 | ΔH-MP | ΔH-S |
| --- | --- | --- | --- | --- | --- |
| ΔANB | β | -1.11 | -0.41 | -0.21 | -0.14 |
|  | *p* | 0.009* | 0.211 | 0.706 | 0.836 |
| ΔSN-MP | β | -0.02 | 0.09 | 0.29 | 0.64 |
|  | *p* | 0.919 | 0.631 | 0.346 | 0.085 |
| Δhead angulation | β | 0.69 | 0.08 | 0.43 | 0.03 |
|  | *p* | 0.000** | 0.136 | 0.000** | 0.760 |
| ΔU1-SN | β | 0.00 | 0.03 | 0.13 | 0.09 |
|  | *p* | 0.966 | 0.393 | 0.027* | 0.213 |
| ΔL1-MP | β | 0.01 | -0.03 | -0.13 | -0.13 |
|  | *p* | 0.843 | 0.440 | 0.057 | 0.115 |
| ΔUvula length | β | 0.00 | 0.18 | 0.11 | 0.48 |
|  | *p* | 0.983 | 0.270 | 0.700 | 0.162 |
| ΔUvula angulation | β | -0.04 | 0.07 | -0.38 | -0.51 |
|  | *p* | 0.645 | 0.343 | 0.005* | 0.002* |

β, beta coefficient, indicates positive or negative correlations between variables of interest.

**p* < 0.05, ***p* < 0.001

∆ indicates pretreatment-to-posttreatment changes in variables of interest.

**Sl Table 3.** Multiple logistic regression of relationships between pharyngeal airway dimensional changes and questionnaire results.

| **Variables** |  | **PSQI** | **SBQ** |
| --- | --- | --- | --- |
| Gender | β | -1.53 | 10.17 |
|  | *p* | 0.201 | 0.999 |
| Age | β | -0.04 | 1.31 |
|  | *p* | 0.645 | 0.999 |
| BMI | β | 0.04 | 1.71 |
|  | *p* | 0.829 | 0.999 |
| Sagittal skeletal pattern | β | 0.96 | -15.01 |
|  | *p* | 0.184 | 0.999 |
| Vertical skeletal pattern | β | -0.06 | 1.02 |
|  | *p* | 0.938 | 1.000 |
| ΔAirway1 | β | 0.07 | 3.54 |
|  | *p* | 0.888 | 1.000 |
| ΔAirway2 | β | -0.58 | -8.41 |
|  | *p* | 0.143 | 0.999 |
| ΔAirway3 | β | 0.19 | 2.49 |
|  | *p* | 0.529 | 1.000 |
| ΔAirway4 | β | -0.06 | 5.10 |
|  | *p* | 0.848 | 0.999 |
| ΔAirway5 | β | 0.09 | -0.36 |
|  | *p* | 0.748 | 1.000 |
| ΔAirway6 | β | 0.22 | 1.63 |
|  | *p* | 0.372 | 1.000 |

β, beta coefficient, indicates positive or negative correlations between variables of interest.

∆ indicates pretreatment-to-posttreatment changes in variables of interest.
